# Remarks on the Stricture of the Urethra of Extreme Calibre

**Published:** 1872-12

**Authors:** 


*Remarks on Stricture of the Urethra of Extreme Calibre, with Cases and a Description of New Instruments for their Treatment.* By F. N. OTIS, M. D. (Reprinted from the New York Medical Journal, February, 1872.)

This is a well written little pamphlet, containing some very valuable ideas concerning stricture of the urethra and its treatment. The new instruments described are most of them known to the profession, but we fear their use is not understood. Prof. Otis illustrates his paper by some well chosen cases, and produces an instructive and valuable paper. Stricture is an accident of no rare occurrence, and all are liable to have cases to treat, and to such as are in doubt as how to proceed, the paper by Dr. Otis will afford valuable suggestions.

